# Supplementary material for: Unveiling new disease, pathway, and gene associations via multi-scale neural network
Source: PLoS One. 2020 Apr 6;15(4):e0231059. doi: 10.1371/journal.pone.0231059 (PMC7135208; doi:10.1371/journal.pone.0231059)
Supplement: S1 File — Project webpage https://life.bsc.es/iconbi/MultiScaleNN/index.html. (PDF) [file pone.0231059.s001.pdf]

Online Supplement: Unveiling new disease, pathway, and gene associations via multi-scale neural networks

Thomas Gaudelet[1], Noël Malod-Dognin [2], Jon Sánchez-Valle [2], Vera Pancaldi[2,3,4], Alfonso Valencia [2,5] and Nataša Pržulj[1,2,5,*]

[1]Department of Computer Science, University College London, London, WC1E 6BT
[2]Barcelona Supercomputing Center (BSC), Barcelona, 08034 Spain
[3]Centre de Recherches en Cancérologie de Toulouse (CRCT), UMR1037 Inserm, ERL5294 CNRS, 31037 Toulouse, France
[4]University Paul Sabatier III, Toulouse, France
[5]ICREA, Pg. Lluís Companys 23, 08010 Barcelona, Spain

1 Supplementary Methods

SI Datasets The base data used in this project is collected from the work of Sanchez-Valle *et al.* [7]. It consists of multiple datasets of gene expressions captured by micro-array technology [9]. The datasets are downloaded from Gene Expression Omnibus (GEO, [1]) and ArrayExpress [2] databases. Each dataset contains measurements from healthy (controls) and affected (patients) subjects. For a given dataset, the measurements originate from bulk samples extracted from the same tissue in each subject. Not all datasets use the same tissue for measurements, as diseases do not necessarily affect the same tissue. Each patient is diagnosed with a single disease. For comparisons, the data is normalized by using the frozen robust multiarray procedure [4] to remove experimental bias. Furthermore, to remove tissue effects, each patient sample is normalised against all the control samples of its original dataset using the Limma method [10]. Up to this point, the data is identical to those used to derive the DMSN network [7]. Then we use the corrected p-values output by Limma to define for each patient a vector of size corresponding to the number of genes and in which the ith entry is equal to 1, -1, or 0 depending on whether the ith gene is significantly (with 5% cutoff) over-, under-, or normally expressed, respectively, for that patient. Additionally, we exclude patients that have no significantly deregulated genes, as we cannot learn anything from them.

The set of diseases is curated by hand for associations with Disease Ontology codes [8], standard ICD9 and ICD10 codes, MeSH terms and OMIM codes [5]. Some of the datasets come from studies investigating subtypes of diseases that are studied by projects linked to other datasets. Based on the number of patients in each study, those datasets were either merged with the more global disease,

or the patients associated with the more global disease were dropped from the study. Specifically, we drop the global disease if the subtype has many more patients associated with it and merge otherwise. Finally, we exclude diseases that have less than 10 associated patients to capture disease heterogeneity in the final dataset and to have sufficient data for each disease to split in a training and testing set.

SI Model A neural network can be expressed as a series of matrix multiplications interleaved with non-linear functions, formally the output $\mathbf{Y}$ of a neural network with $n-1$ hidden layers can be written as $\mathbf{Y} = f_n\left(\mathbf{W}_n f_{n-1}\left(\dots f_1((\mathbf{W}_1\mathbf{X})\right)\right)$ where $\mathbf{X}$ represents the input data, $\mathbf{W}_i$ the weights of layer i, and $f_i(\cdot)$ the non-linear function applied to the output of the ith layer. The optimization problem can be written as the minimization of the loss function $\mathcal{L} = g\left(\hat{\mathbf{Y}}, \mathbf{Y}\right)$, where $\hat{\mathbf{Y}}$ is the objective, or ground truth, and $g(\cdot)$ is a predefined function. Multinomial logistic regression (MLR) and the proposed GDP architecture can be written as

$$\mathbf{Y}^1 = s\left(\mathbf{W}^1\mathbf{X}\right) \tag{1}$$

$$\mathbf{Y}^2 = s\left(\mathbf{W}_2^2 \tanh\left(\mathbf{W}_1^2\mathbf{X}\right)\right) \tag{2}$$

where s is the softmax function, typically used for multiclass classification problems and tanh denotes the hyperbolic tangent. Matrices $\mathbf{X}$ and $\hat{\mathbf{Y}}$ represent our data. Each column of $\mathbf{X}$ corresponds to the differential gene expression of a patient, and each column of $\hat{\mathbf{Y}}$ corresponds to a patient's diagnosis (the prediction of which is the objective of the framework). $\mathbf{W}^1 \in R^{n_d \times n_g}$ and $\mathbf{W}_2^2 \in R^{n_d \times n_p}$ correspond to fully-connected layers. The layer corresponding to $\mathbf{W}_1^2 \in R^{n_c \times n_g}$ represents biological pathway membership of the genes, i.e. the trainable weights of the matrix correspond only to entries (i, j) where gene j is part of the ith protein complex.

SI Methods Formally, the local variations δf of a single-argument function f due to a change $\delta x = x - x_0$ in input can be approximated with the first order Taylor expansion as

$$\delta f(x) = \frac{df}{dx}(x_0)\delta x + O(x^2).$$

Thus, the magnitude of the local variations of f with respect to perturbation δx from x_0 is given by $|\frac{df}{dx}(x_0)|$. Based on this approximation, we extract from each neural network a score between an entity represented by a unit of the neural network (e.g, a pathway, or a gene) and each disease (output unit). Specifically, for a neural network NN, we denote $\mathrm{nn}^i : [0,1]^{n_i} \mapsto R^{n_o}$ the function corresponding to the operation of a neural network NN from the n_i outputs of layer i to the final n_o logits of the neural network, i.e. scores before softmax. E.g., for GPD, we have $\mathrm{nn}_2^1(\mathbf{x}) = \mathbf{W}_2^2 \tanh\left(\mathbf{W}_1^2\mathbf{x}\right)$. Then, the association score $s_{i,j,k}$ between the jth output unit of layer i, denoted u_j^i, of neural network NN, and disease k is given by

$$s_{i,j,k} = \left| \left[\frac{\partial \mathrm{nn}^i}{\partial u_j^i}(\mathbf{x_0}) \right]_k \right|,$$

where the reference point is chosen as the null vector, $\mathbf{x_0} = \mathbf{0}$, which corresponds implicitly to a healthy state in our formulation.

SI Metrics The cross-entropy loss (CLE) of a classifier is defined as

$$\text{CLE} = \frac{1}{m} \sum_{i=1}^{m} \sum_{j=1}^{n} -y_{ij} \log(\hat{y}_{ij}),$$

where m represents the number of samples (patients), n the number of classes (diseases), y_{ij} indicates if patient i is diagnosed with disease j (1 if true 0 otherwise), and $\hat{y}_{ij}$ is the jth output value of the classifier for patient i. A relatively small CEL means that the output probability distribution of a classifier is closer to the deterministic one-hot encoding of the true labeling, i.e. the classifier gives a higher probability to the true class and a very small probability to the other classes.

The micro-averaged precision (Pre_μ) of a classifier gives a measure of the overall precision of the classifier and is defined as

$$\text{Pre}_\mu = \frac{tp}{m},$$

where tp corresponds to the number of accurately classified patients and m represents the number of patients.

The macro-averaged precision (Pre_M) of a classifer gives an average of the precision across the different classes (diseases) and is defined as

$$\text{Pre}_M = \frac{1}{n} \sum_{i=1}^{n} \frac{tp_i}{m_i},$$

where tp_i corresponds to the number of accurately classified patients for disease i and m_i represents the number of patients diagnosed with the same disease. Pre_M can be more informative than Pre_μ when considering the problem with class imbalance.

SI Baselines The Frequency of Differential Expression (FDE) score of a disease–gene association corresponds to how frequently that gene is consistently differentially expressed in patients having the disease, i.e., for disease d and gene g, the association score, s_{dg}, is given by

$$s_{dg} = \left| \frac{1}{|\mathcal{P}_d|} \sum_{p \in \mathcal{P}_d} \mathbf{X}_{gp} \right|, \tag{3}$$

where we amalgamate entities (disease, gene, and patient) with their indices, $\mathcal{P}_d$ denotes the set of patients having disease d, and $\mathbf{X}$ corresponds to the data matrix introduced in Methods.

The Katz method uses disease specific Protein–Protein Interaction (PPI) network, where each node of a standard PPI network is associated to a score (here the FDE of each gene for the disease

considered). The authors then use Katz-centrality on each disease PPI network to extract a final score for each disease–gene association (here we use the absolute value). The higher the score, the higher the association is expected to be true. We download the PPI data from BioGRID [6] and IID [3] and create our PPI network from the union of both databases restricted to our set of genes. Finally, we perform a grid-search to identify the best parameters for the model by trying to maximise the area under the precision-recall curve metric.

2 Supplementary figures and tables

Disease Name	Patients Count	Disease Name	Patients Count
non-small cell lung carcinoma	490	amyotrophic lateral sclerosis	36
oral cavity cancer	248	juvenile myelomonocytic leukemia	34
psoriasis	223	nasopharynx carcinoma	31
myelodysplastic syndrome	187	sarcoidosis	30
bacterial sepsis	181	dermatomyositis	29
colorectal cancer	154	myositis	29
asthma	138	cervical cancer	28
mature T-cell and NK-cell lymphoma	131	multiple sclerosis	27
alzheimers disease	128	turner syndrome	26
kidney cancer	121	interstitial lung disease	25
schizophrenia	114	multiple myeloma	22
chronic obstructive pulmonary disease	89	type 2 diabetes mellitus	20
pilocytic astrocytoma	79	essential thrombocythemia	19
thyroid cancer	79	sjogrens syndrome	19
bladder carcinoma	79	jobs syndrome	18
cerebrovascular disease	78	sotos syndrome	18
adrenocortical carcinoma	77	oral mucosa leukoplakia	17
uremia	75	rhabdoid cancer	17
endometriosis	74	dengue disease	17
major depressive disorder	67	esophagus squamous cell carcinoma	17
irritable bowel syndrome	65	ulcerative colitis	17
stomach cancer	65	anogenital venereal wart	16
oligodendroglioma	64	alcoholic hepatitis	15
systemic lupus erythematosus	61	campylobacteriosis	14
hepatocellular carcinoma	59	spondylosis	14

myocardial infarction	57	vitiligo	14
breast cancer	57	mitochondrial metabolism disease	14
malignant pleural mesothelioma	55	osteosarcoma	14
glioblastoma multiforme	53	cornelia de lange syndrome	14
acute myeloid leukemia	52	aphthous stomatitis	13
autistic disorder	51	sinusitis	13
hcv infection	49	sickle cell anemia	13
hepatoblastoma	49	atrial fibrillation	13
pancreatic ductal adenocarcinoma	46	hepatitis b	12
prostate cancer	46	peripheral vascular disease	12
ovarian cancer	43	acne	12
monoclonal gammopathy of undetermined significance	43	crohns disease	11
medulloblastoma	41	leishmaniasis	11
polycythemia vera	41	follicular lymphoma	10
atopic dermatitis	40	myelofibrosis	10
trachoma	39	leigh disease	10
rosacea	38		

Supplementary Table 1: Cohort size for each disease in the dataset.

hyperparameter	10	0.1	0.01	0.001	0
L1-regularization	2026 ± 4.3	24.5 ± 0.046	6.39 ± 0.003	2.71 ± 0.047	$\mathbf{1.09 \pm 0.066}$
L2-regularization	$4.42 \pm 4.8e^{-7}$	4.38 ± 0.012	3.48 ± 0.032	2.02 ± 0.026	/
dropout ratio	0.25	0.5	0.75	0.9	0
dropout	1.14 ± 0.121	1.10 ± 0.130	1.12 ± 0.086	1.10 ± 0.081	/

Supplementary Table 2: Results of cross-validation to fix regularisation hyperparameters (L1-, L2-, or dropout regularisations). The scores correspond to cross-entropy loss. The best results are obtained with no regularisation (score in bold).

References

[1] Tanya Barrett et al. Ncbi geo: archive for functional genomics data sets—update. *Nucleic Acids Research*, 41(D1):D991–D995, 2013.

[2] Nikolay Kolesnikov et al. Arrayexpress update—simplifying data submissions. *Nucleic Acids Research*, 43(D1):D1113–D1116, 2015.

[3] Max Kotlyar et al. Integrated interactions database: tissue-specific view of the human and model organism interactomes. *Nucleic Acids Research*, 44(D1):D536–D541, 2015.

[4] Matthew N McCall et al. Frozen robust multiarray analysis (frma). *Biostatistics*, 11(2):242–253, 2010.

[5] Victor A McKusick. *Mendelian inheritance in man: a catalog of human genes and genetic disorders*, volume 1. JHU Press, 1998.

[6] Rose Oughtred et al. The biogrid interaction database: 2019 update. *Nucleic acids research*, 47(D1):D529–D541, 2018.

[7] Jon Sánchez-Valle et al. Unveiling the molecular basis of disease co-occurrence: towards personalized comorbidity profiles. *bioRxiv, doi:10.1101/431312*, 2018.

[8] Lynn Marie Schriml et al. Disease ontology: a backbone for disease semantic integration. *Nucleic Acids Research*, 40(D1):D940–D946, 2011.

[9] Almut Schulze et al. Navigating gene expression using microarrays—a technology review. *Nature Cell Biology*, 3(8):E190, 2001.

[10] Gordon K Smyth. Limma: linear models for microarray data. In *Bioinformatics and Computational Biology Solutions using R and Bioconductor*, pages 397–420. Springer, 2005.

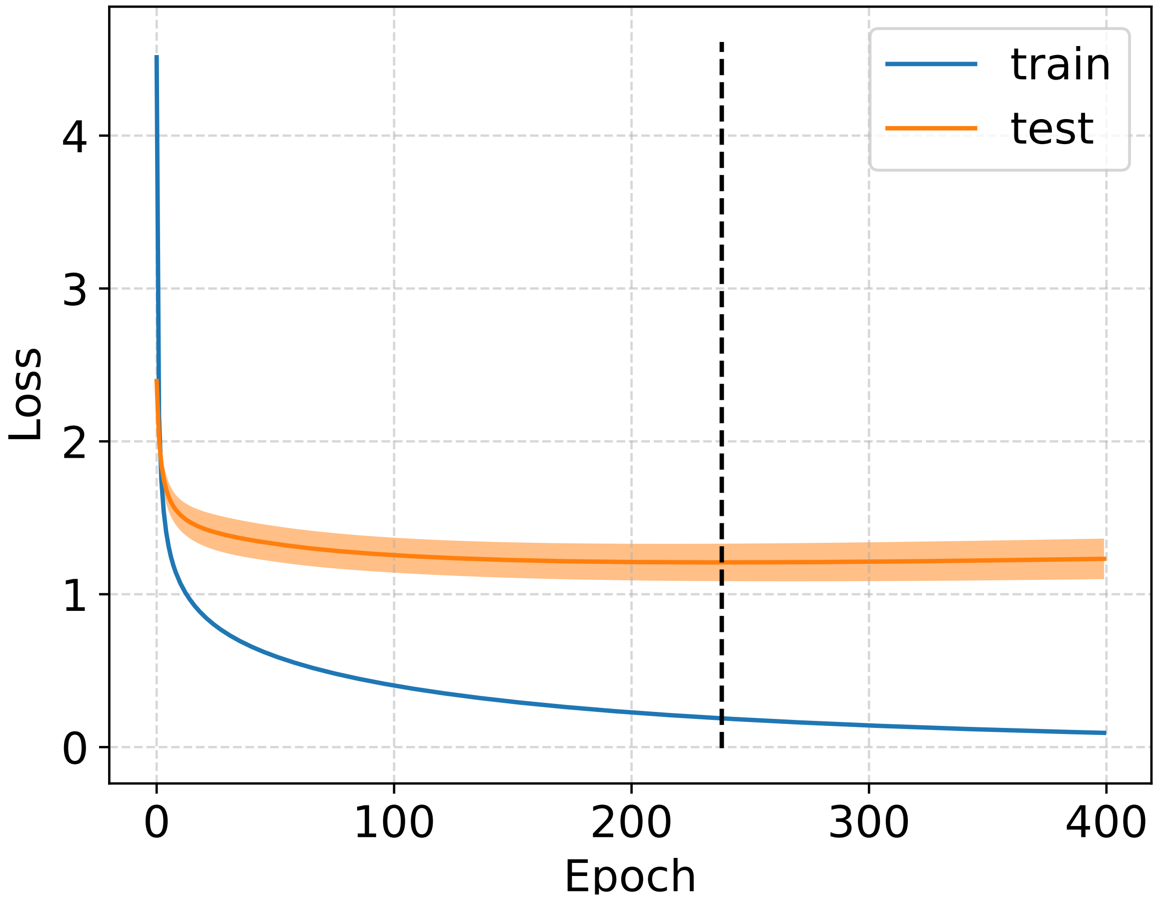

Supplementary Fig. 1: Train and test loss curve for the MLR model with respect to the number of epochs during the cross-validation. The vertical black line indicates the number of epochs which give the lowest loss.

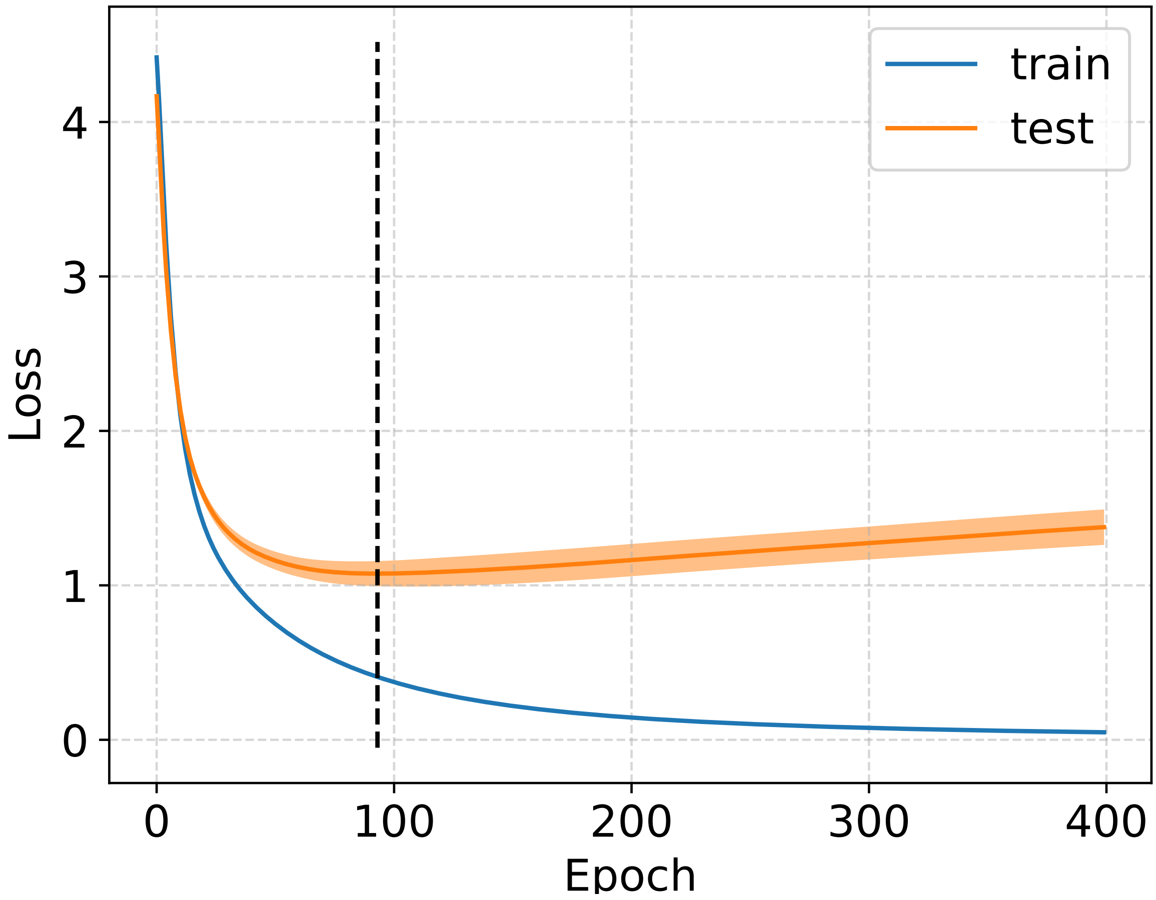

Supplementary Fig. 2: Train and test loss curve for the GDP model with respect to the number of epochs during the cross-validation. The vertical black line indicates the number of epochs which give the lowest loss.

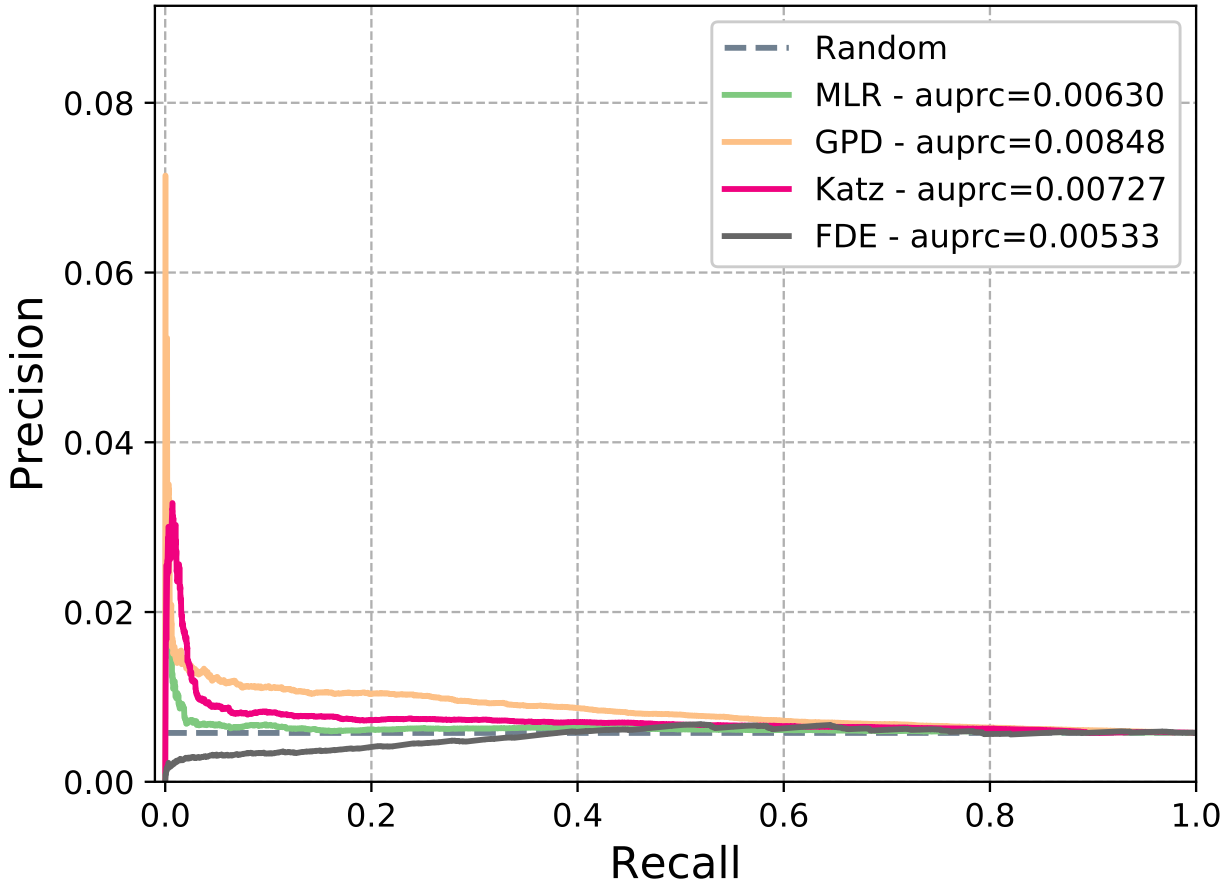

Supplementary Fig. 3: Precision-recall curve of our predictions for disease–genes associations.

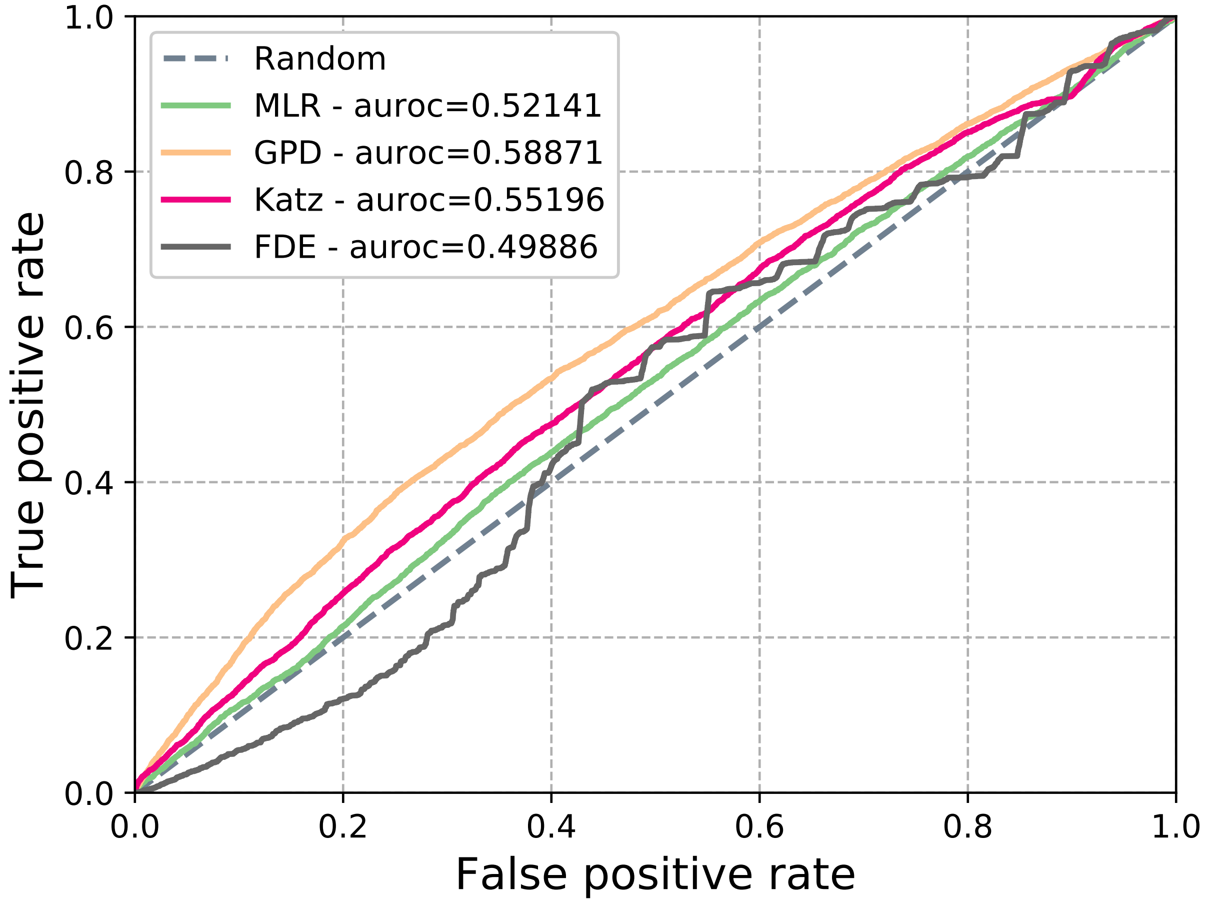

Supplementary Fig. 4: ROC curve of our predictions for disease–genes associations.

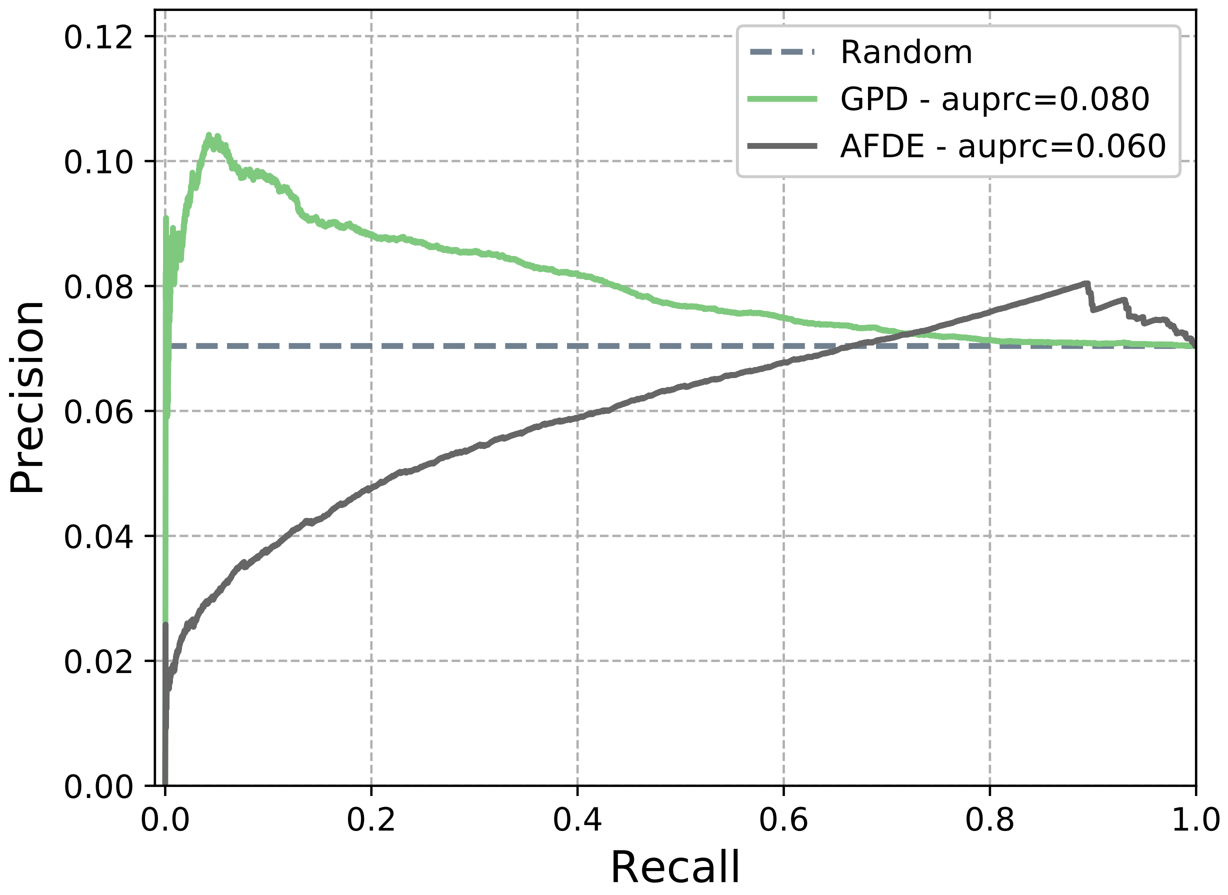

Supplementary Fig. 5: Precision-recall curve of our disease–pathways associations predictions.

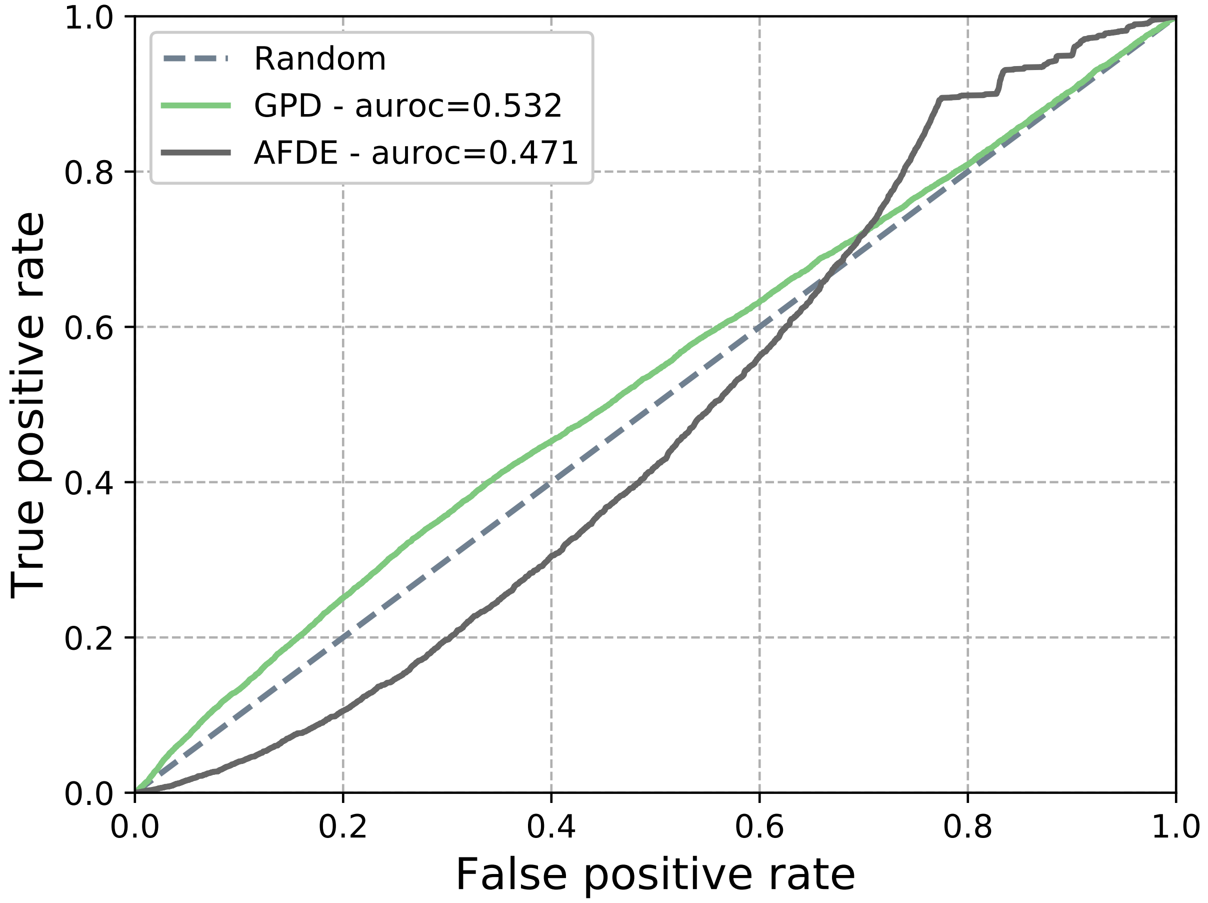

Supplementary Fig. 6: ROC curve of our disease–pathways associations predictions.
