# Supplementary material for: Unveiling new disease, pathway, and gene associations via multi-scale neural network
Source: PLoS One. 2020 Apr 6;15(4):e0231059. doi: 10.1371/journal.pone.0231059 (PMC7135208; doi:10.1371/journal.pone.0231059)
Supplement: Attachment — Submitted filename: Response to Reviewers.pdf [file pone.0231059.s002.pdf]

The 12th of February, 2020

Dear Editor,

Thank you for inviting us to submit a revised version of our manuscript titled “Unveiling new disease, pathway, and gene associations via multi-scale neural network”.

We revised our paper to address the reviewers’ suggestions. Our responses to the reviewers’ comments are summarized below.

We believe that the manuscript is now suitable for publication in PloS One, and look forward to hearing from you.

Sincerely yours,

Nataša Pržulj

Reviewer 1:

1. In the introduction section, line 20: “In specific cancers, Abeel et al. [6] use support vector machines and ensemble feature selection methods to select putative gene biomarkers.”
You might consider also to refer to articles that show methods that can work on different omic signatures for cancer in general like Ciucci et al., 2017 Scientific reports.
Response: We followed the suggestion of the reviewer and in our revised manuscript we updated the introduction accordingly (page 2, paragraph 1).

2. The authors gave an association score for the prediction of disease-disease, disease-pathway and disease-gene relationships, which was lately use to obtain the highest 10 predictions under the three mentioned relationships, validating the predictions by literature search. It would be interesting to known the extent to which this score is “high-enough” to obtain valuable information from the trained GPD model, since this score magnitude is not mentioned on the manuscript.
Response: We thank the reviewer for the comment. There is no out-of-the-box threshold that can answer this question and we chose here to validate our scoring strategy with manual literature curation and precision-recall analysis against an independent ground-truth. However, one could use the precision-recall analysis we ran as a way to estimate how many of the top scoring associations can be considered relevant. We adjusted the main to reflect this. (page 4, paragraph 5)

Reviewer 2:

1. The authors should clarify their method for the prediction of disease-(disease/pathway/gene) relationships. What is meant with the intensity of local variation of d with respect to perturbation of u? Are there thresholds involved, e.g., when is the intensity high enough to create the relationship.
Response: We thank the reviewer for the comment. The score used to identify molecular links to the disease rests on the analysis of variations of the output of a function (e.g., a neural network or part of one) with respect to the variation of one of its input (e.g., gene expression). Intuitively, we assume that the trained neural network links a gene, for instance, to a disease if a change in the expression of the gene leads to a variation in the disease score given by the output of the neural network. Effectively, this is the analytical equivalent of manually varying the expression of each gene and measuring the impact on the score and prediction of each disease. We postulate here that the higher the induced variation, the more likely there is an actual biological connection between the gene and disease. To assess this, we rank disease-gene pairs based on this score and we evaluate how well this ranking captures known interactions with precision-recall and receiver operating characteristic analysis as well as manual literature curation. We updated the manuscript to clarify those points. (page 4, paragraph 5)

The second part of the comment relating to threshold is the same as the second comment of the first reviewer and we refer the reader to the response above.

2. Regularization: The authors utilize early stopping, but no other regularization. Here, particular considering the number of genes vs. the number of samples might pose a problem. Have the authors considered heavy drop-out regularization of the input layer, which might boost the necessity to use the pathway layer since it cannot any longer rely on the presence of single genes?
Response: We thank the reviewer for the suggestion. We had investigated the addition of L2 and L1 regularisations with a cross-validation but observed that the models without were performing better in terms of cross-entropy loss thus we removed any. Heavy dropout on the input layer was not investigated, following the reviewer suggestion, we have run test with it and observed that similarly it was not leading to improvements in terms of cross-entropy loss or overfitting. We updated the text to mention the tests with addition of L1, L2, or dropout regularisations and added a supplementary table with cross-validation scores for each regularisation. (page 4, paragraph 3 and Supplementary Table 2)

3. Connected to the point above, the authors should depict the learning behavior of their network wrt. the loss training vs. validation. Is the observed behavior expected, or does it indicate over- or underfitting.
Response: We followed the reviewer suggestion, and in our revised manuscript we show the learning behaviour of the neural networks (Supplementary Figures 1 and 2). Indeed, the plots suggest some overfitting, however, as mentioned above, the performances remain better than when regularisation is added to address the overfitting.
